# Increase in Healthcare-Associated Infections: Is COVID-19 Responsible? A Narrative Review

**DOI:** 10.3390/microorganisms14091952

**Published:** 2026-09-03

**Authors:** Pietro Crispino, Antonello Viceconti, Valentina Camardo

**Affiliations:** 1 Dipartimento di Medicina Interna, Ospedale di Latina, ASL Latina, 04100 Latina, Italy; 2 Pronto Soccorso, Ospedale di Lagonegro, AOR San Carlo, 85042 Lagonegro, Italy; 3 Unità di Ostetricia e Ginecologia, Ospedale di Lagonegro, AOR San Carlo, 85042 Lagonegro, Italy

**Keywords:** COVID-19, nosocomial infection, assistance

## Abstract

During the COVID-19 pandemic, healthcare-associated infections (HAIs) showed a significant increase, exacerbated by the prevalence of multidrug-resistant (MDR) organisms. Antibiotic overuse and infection control practices influenced HAI incidence rates, alongside the rising prevalence of resistant pathogens such as methicillin-resistant Staphylococcus aureus. Antibiotic-resistant pathogens causing healthcare-associated infections in COVID-19 patients include—in addition to methicillin-resistant *Staphylococcus aureus*—metallo-β-lactamase-producing carbapenem-resistant *Enterobacteriaceae*, carbapenem-resistant *Acinetobacter baumannii*, extended-spectrum β-lactamase-producing *Klebsiella pneumoniae*, and vancomycin-resistant *enterococci*. COVID-19 impacted bacterial healthcare-associated infections in various ways, with an increase in the incidence of metallo-β-lactamase-producing, carbapenem-resistant organisms—a trend already noted prior to the pandemic. Furthermore, the pandemic laid the groundwork for future challenges in HAI management, highlighting the need for rigorous infection prevention and control protocols. Poorer outcomes were observed in hospitalized COVID-19 patients with antibiotic-resistant infections. Although increased infection prevention and control (IPC) measures led to a reduction in certain site-specific hospital-acquired infections, in other contexts, COVID-19 was associated with an increased incidence of bacterial hospital-acquired infections. Further research is needed to determine the cost–benefit ratio of maintaining COVID-19-related infection prevention and control protocols beyond the pandemic in order to reduce the impact of hospital-acquired infections. Furthermore, it is necessary to assess the long-term impact of the high usage of certain broad-spectrum antibiotics during the COVID-19 pandemic.

## 1. Background

In the early stages of the COVID-19 pandemic, data on bacterial infections in COVID-19 patients were limited. However, Guan et al. had already hypothesized a link between the viral infection and a potential rise in healthcare-associated infections (HAIs) [1]. Unfortunately, early in the pandemic, there was a delay in conducting thorough bacteriological or fungal assessments of hospitalized patients, as healthcare efforts were primarily focused on curbing the virus’s spread and treating cases of pneumonia complicated by severe respiratory failure [2,3]. Zhou et al. [4] found that sepsis was the most frequently observed complication in both survivors and non-survivors of COVID-19. The emergence of the COVID-19 pandemic placed immense strain on global healthcare systems and led to a marked increase in healthcare-associated infections (HAIs). Recent studies indicate that bacterial infections in COVID-19 patients—particularly those with severe forms of the disease—have become a new area of concern. Hospital-acquired infections caused by antibiotic-resistant pathogens, such as methicillin-resistant *Staphylococcus aureus* (MRSA) and extended-spectrum β-lactamase (ESBL)-producing *Klebsiella pneumoniae*, were reported with increasing frequency during the pandemic [4]. The authors [5] noted that, in these patients, superimposed sepsis could stem from the preceding viral infection; specifically, a study by Zhang G et al. [5] observed that COVID-19 patients—particularly those admitted to the intensive care unit (ICU) and undergoing mechanical ventilation—exhibited a significantly higher rate of bacterial co-infection compared to patients with non-severe COVID-19. Another study conducted in the United Kingdom reported a low frequency of bacterial co-infection in patients treated early for COVID-19, demonstrating that the more severe the viral infection, the greater the risk of contracting HAIs [6]. Subsequent findings began to reveal the specific bacterial pathogens causing HAIs in COVID-19 patients, showing that *Streptococcus pneumoniae* and *Hemophilus influenzae*, in particular, were associated with community-acquired pneumonia, while *Staphylococcus aureus* was linked to both community-acquired co-infection (community-acquired pneumonia) and hospital-associated superinfections (ventilator-associated pneumonia and hospital-acquired pneumonia) [7]. Community- and hospital-acquired urinary tract infections were caused by *Escherichia coli*, *Klebsiella pneumoniae*, and *Enterobacter faecium* [7]. A recent analysis conducted in the United States reported that *Staphylococcus aureus* was more frequently detected in pharyngeal and oropharyngeal swabs from COVID-19-positive patients compared to SARS-CoV-2-negative individuals [8]. This evidence contributed to a substantial rise in antibiotic use during the pandemic—driven largely by empirical prescribing due to fears of secondary infections in COVID-19 patients, particularly those critically ill in intensive care—which led to increased antimicrobial resistance and, as highlighted in the study by Spigaglia, a rise in *Clostridioides difficile* cases [9,10]. However, data collected throughout the pandemic years indicated not only an increase in healthcare-associated infections (HAIs) [11] but also significant improvements in infection control measures; specifically, mandatory personal protective equipment (PPE) played a crucial role in reducing hospital-acquired infections during both the initial stages and the subsequent spread of COVID-19 variants [12]. The introduction of COVID-19 vaccines played a pivotal role in limiting healthcare-associated infections, particularly among high-risk population groups. Several studies have demonstrated that mRNA vaccines provided effective protection against symptomatic and severe infection, significantly reducing the risk of hospitalization and lowering the incidence rate of post-vaccination infections [13,14]. In contrast to vaccines, the role of direct-acting antivirals against COVID-19 in preventing HAIs has been relatively limited, as most studies focused on their utility in treating the disease rather than on preventing infections [15]. Monoclonal antibodies (mAbs) have been effectively used to treat COVID-19, demonstrating a reduction in healthcare-associated infections, particularly among vulnerable populations [16,17,18,19]. Early use of mAbs in high-risk COVID-19 patients has led to a significant reduction in hospitalizations and mortality [16,17,18,19].

Therefore, the aim of this review is to evaluate the literature available to date regarding the urgent need to update antimicrobial policies and improve strategies for the prevention and control of healthcare-associated infections (HAIs)—which represent a further test for healthcare systems following the pandemic—while considering the implications for the future efficacy of available antibiotics and the persistent role of climate change and its long-term public health implications [20].

## 2. Search Strategy and Methods

A comprehensive literature search was performed in PubMed/MEDLINE, Scopus, and Web of Science databases for articles published between January 2020 and mid-2026. Search terms included combinations of Medical Subject Headings (MeSH) and free-text keywords: “COVID-19”, “SARS-CoV-2”, “healthcare-associated infections”, “nosocomial infections”, “antimicrobial resistance”, “Klebsiella pneumoniae”, “antiviral therapy”, “vaccines”, and “monoclonal antibodies”. Original research articles, systematic reviews, meta-analyses, and clinical guidelines published in English and Italian were screened and evaluated for inclusion based on relevance to HAI incidence, AMR dynamics, and clinical management in COVID-19 patients.

## 3. The Role of COVID-19 in Healthcare-Associated Infections

COVID-19 has radically altered the global healthcare landscape, introducing new challenges in the management of healthcare-associated infections. A review highlighted the prevalence of multidrug-resistant bacteria and fungi in patients with SARS-CoV-2 infections, underscoring how hospital-acquired infections are becoming an increasing problem, particularly among those requiring hospitalization for COVID-19 (Table 1). Common resistant pathogens include MRSA and NDM-producing Enterobacterales, highlighting the need for rigorous infection control [21].

Furthermore, the impact of COVID-19 on HAIs is not limited to bacterial infections. Spigaglia [9], consistent with an earlier study of ours [22], reported an increase in *Clostridioides difficile* infections following the pandemic, suggesting that hospital containment capabilities were strained by the restructuring of standard operating procedures and the excessive use of antibiotics to treat potential co-infections. A systematic review of 37 studies revealed that certain HAIs saw a significant increase during the COVID-19 period, alongside a higher incidence of resistant bacterial strains [23]. Conversely, some reports have suggested that emerging control measures—such as improved hygiene practices and close monitoring of at-risk patients—led to a decrease in other associated infections, such as MRSA [21]. The relationship between hospital-acquired infections and COVID-19 is complex. Various publications have highlighted the growing concern regarding the pandemic’s impact on the survival of patients with HAIs; co-infection has emerged as an adverse prognostic factor, significantly increasing mortality rates among COVID-19 patients. The pandemic also drove an increase in antimicrobial use (Table 2) [11]. However, this trend has led to rising antimicrobial resistance, prompting experts to recommend rigorous stewardship policies to prevent the spread of antibiotic-resistant organisms. Medication management guidelines for COVID-19 patients are currently being re-evaluated to ensure that therapies are both appropriate and effective [20] (Table 2). ESBL-positive *Klebsiella pneumoniae* infection has been associated with significant mortality in COVID-19 patients, with mortality rates of 57% and 69.6% at 14 and 30 days, respectively. In a systematic study, the *Klebsiella pneumoniae* co-infection rate among critically ill COVID-19 patients was 42.91%, while the overall incidence of bacterial infections in these patients was estimated at 15.5%. Furthermore, the rational use of antibacterial drugs has become crucial given the emergence of carbapenem-resistant strains. Therefore, *Klebsiella pneumoniae* co-infection and mortality dynamics require special attention in the context of the pandemic. The incidence and mortality associated with ESBL-positive *Klebsiella pneumoniae* infections in COVID-19 patients are of significant concern, characterized by high mortality rates and a high prevalence of co-infections among hospitalized patients.

A study analyzing 157 clinical trials found that 30-day mortality rates for *Klebsiella pneumoniae* were 29% overall and higher among patients admitted to intensive care, particularly those with carbapenem-resistant *Klebsiella pneumoniae* (CRKP) infections [24]. Furthermore, patients with *Klebsiella pneumoniae* infections who have cardiovascular and pulmonary comorbidities face a higher risk of mortality; a 42.91% rate of *Klebsiella pneumoniae* co-infection was reported among critically ill patients with COVID-19 [25]. A large observational study in Italy specifically found that 48.3% of patients with hypervirulent *Klebsiella pneumoniae* infections and COVID-19 died within 30 days of diagnosis [26]. This not only highlights the high mortality associated with *Klebsiella pneumoniae* infections but also underscores the role of antibiotic resistance in worsening clinical outcomes. From an epidemiological perspective, the emergence of resistant bacteria during the pandemic has further complicated treatment and infection control dynamics, making the rational use of antibiotics essential [27]. This landscape is further complicated in COVID-19 patients, with increased sepsis rates in both hospital settings and intensive care units. A review article on sepsis epidemiology highlights that sepsis mortality rates in critically ill COVID-19 patients are significantly higher, exacerbated by the onset of secondary infections or co-infections [28]. Although traditionally considered exclusively a hospital-acquired pathogen, multidrug-resistant (MDR) *Klebsiella pneumoniae* is also emerging in the community, yet its population structure and dissemination remain unknown. A recent genomic survey of 2006 ceftriaxone-nonsusceptible outpatient isolates from 42 US states reveals that the spread of resistance is primarily driven by the CTX-M-15 gene—an extended-spectrum beta-lactamase (ESBL)—present across various *Klebsiella pneumoniae* lineages [29]. This gene, carried by a family of IncFIB (Kpn3) plasmids, has spread to hundreds of lineages, combining antibiotic resistance with stress and metal tolerance traits that may enhance survival outside the human host. The majority of isolates originated from elderly women with urinary tract infections, for whom oral treatment options are becoming increasingly ineffective. Capsule and O-antigen profiling reveals extensive antigenic diversity; however, a manageable subset of serotypes could offer broad coverage for vaccine development. These findings highlight a rapidly evolving, plasmid-driven epidemic of community-acquired MDR *Klebsiella pneumoniae* [29].

### 3.1. Mortality from Klebsiella pneumoniae and COVID-19

A meta-analysis comprising 157 studies revealed that 30-day mortality for patients with *Klebsiella pneumoniae* disease is 29%. This figure rises in various settings; notably, patients with hospital-acquired infections face poorer outcomes and require more aggressive monitoring in intensive care units [24]. Associated mortality is exacerbated by the presence of resistant pathogens—particularly CRKP strains, which have seen a significant increase over time—highlighting the need for decisive public health interventions to combat the antimicrobial crisis. A systematic analysis highlighted the phenomenon of co-infection, reporting that 42.91% of critically ill COVID-19 patients presented with *Klebsiella spp. co-infections*, with *Klebsiella pneumoniae* predominating [25]. A high proportion of these patients required aggressive antibiotic therapy, and an estimated 32.5% of those with co-infections died, demonstrating the severity of the associated risk [25]. The emergence of carbapenem-resistant *Klebsiella pneumoniae* is particularly concerning, as antibiotic use in these cases has been linked to 14-day and 30-day mortality rates of 57% and 69.6%, respectively. Furthermore, seroprevalence was driven primarily by OXA-48 and NDM, necessitating rigorous and timely microbiological surveillance [30]. Sepsis is a condition that has increased significantly in the context of COVID-19. Recent research indicates that identifying sepsis in COVID-19 patients proved challenging during the pandemic and suggests that current sepsis management practices may require adaptation to better detect bacterial infections such as *Klebsiella pneumoniae* [28,31].

### 3.2. Pathogen-Level Analysis of Healthcare-Associated Infections (HAIs) During the COVID-19 Pandemic

The landscape of healthcare-associated infections (HAIs) underwent a profound shift during the COVID-19 pandemic. While early reports heavily emphasized Klebsiella pneumoniae as a primary cause of secondary infections, a comprehensive analysis reveals a multifactorial surge across a broad spectrum of multidrug-resistant (MDR) bacterial and opportunistic fungal pathogens. This phenomenon was driven by unprecedented intensive care unit (ICU) overcrowding, prolonged mechanical ventilation, widespread empirical antimicrobial use, and critical disruptions in standard infection control protocols [32,33,34].

#### 3.2.1. Multidrug-Resistant (MDR) Bacterial Pathogens

- Methicillin-Resistant *Staphylococcus aureus* (MRSA) and Other Staphylococci

The incidence of MRSA and coagulase-negative staphylococci escalated sharply in COVID-19 cohorts. Patients with severe SARS-CoV-2 often experienced prolonged endothelial damage and required central venous catheters, significantly elevating the risk of catheter-associated bloodstream infections (BSI). The systematic overuse of broad-spectrum empirical antibiotics upon admission accelerated selection pressure, cementing MRSA as a dominant Gram-positive culprit in hospital-acquired pneumonias and secondary bacteremia [32,33,34,35,36].

- *Pseudomonas aeruginosa*

As an opportunistic pathogen heavily associated with biofilms, Pseudomonas aeruginosa emerged as a major cause of ventilator-associated pneumonia (VAP) in COVID-19 intensive care units. Critical shortages of nursing staff and high patient-to-staff ratios compromised routine patient repositioning, subglottic suctioning, and ventilator circuit maintenance. These operational deficits facilitated the colonization and persistence of MDR P. aeruginosa strains within respiratory tracts and moist hospital environments [32,33,34,35,36].

- *Acinetobacter baumannii*

*Acinetobacter baumannii* represented one of the most severe antimicrobial resistance challenges during the pandemic. Highly resilient to environmental desiccation, MDR and carbapenem-resistant A. baumannii (CRAB) caused extensive outbreaks in dedicated COVID-19 ICUs. Overburdened environmental cleaning services and the rapid turnover of critically ill patients allowed CRAB to colonize medical equipment, leading to high-mortality outbreaks of VAP and bloodstream infections [32,33,34,35,36,37].

- Other Clinically Relevant MDR Enterobacterales

Beyond K. pneumoniae, other Gram-negative bacilli such as Escherichia coli and Enterobacter species exhibited significant increases in extended-spectrum beta-lactamase (ESBL) production and carbapenem resistance. This shift was heavily correlated with the disruption of active surveillance screening programs and a general collapse of contact isolation precautions due to emergency cohorting practices [32,33,34,35,36,37].

#### 3.2.2. Fungal Healthcare-Associated Infections

The burden of nosocomial fungal infections was vastly amplified during the pandemic, shifting from a niche clinical concern to a major driver of ICU mortality [36,37]. Unlike traditional Candida species, C. auris exhibits an exceptional capacity for healthcare-associated, patient-to-patient transmission due to its high persistence on abiotic surfaces, medical equipment, and skin niches.

- *Candida auris*

The pandemic acted as a major catalyst for the global dissemination of Candida auris, an emerging, highly transmissible, and frequently pan-antifungal-resistant yeast [37,38,39,40]. Outbreaks were specifically concentrated within dedicated COVID-19 hospital units, driven by unique operational pressures:

PPE Shortages and Reuse: To conserve supplies during shortages, healthcare facilities implemented the extended wear or reuse of personal protective equipment (PPE) or utilized underlayers of gowns and gloves. This unintended practice turned healthcare workers’ attire into vectors, carrying the resilient fungus between cohorted patients. Altered Disinfection Protocols: The heavy focus on neutralizing airborne SARS-CoV-2 often led to a reliance on disinfectants that were ineffective against C. auris spores, which require specialized sporicidal agents or chlorine-based products to eradicate them from shared medical equipment [37,38,39,40].

Lapses in Screening and Hygiene: Hand hygiene compliance frequently deteriorated under severe staff burnout, while active mycological screening programs were suspended to alleviate laboratory burdens, allowing C. auris to spread undetected among severely ill, immunocompromised individuals [37,38,39,40].

- COVID-19-Associated Mucormycosis (CAM)

COVID-19-associated mucormycosis (CAM) emerged as a catastrophic opportunistic fungal infection, particularly noted in specific geographic regions during devastating pandemic waves [41,42,43]. The intersection of specific physiological and clinical risk factors accelerated its incidence:

Corticosteroid-Induced Immunosuppression: The standard administration of high-dose corticosteroids (e.g., dexamethasone) to mitigate COVID-19 hyperinflammation severely impaired macrophage and neutrophil function, which are the body’s primary defense lines against fungal spores [41,42,43].

Hyperglycemia and Uncontrolled Diabetes: Corticosteroid therapy induced profound secondary hyperglycemia. In patients with pre-existing, poorly controlled diabetes mellitus or diabetic ketoacidosis (DKA), elevated blood glucose and subsequent metabolic acidosis created an optimal environment for Mucorales germination. Acidosis disrupts transferrin binding, releasing free iron into the serum, which acts as a vital growth catalyst for these fungi [41,42,43].

Severe Tissue Hypoxia: The extensive endothelial damage, microvascular thrombosis, and severe alveolar hypoxia characteristic of advanced COVID-19 resulted in localized tissue necrosis, providing an ideal ischemic substrate for angioinvasive fungal hyphae to proliferate [41,42,43].

In dedicated COVID-19 hospital units, several specific breakdown factors directly contributed to C. auris outbreaks, including critical changes in infection-control practices, severe shortages that led to the extended wear or reuse of personal protective equipment (PPE), and the sub-optimal deployment of environmental disinfection protocols. Furthermore, the reduction or suspension of routine microbiological screening programs allowed this highly transmissible and often pan-antifungal-resistant pathogen to spread undetected among severely ill, hospitalized patients, posing a massive systemic challenge to international healthcare facilities [38,39,40,41,42,43,44].

### 3.3. Impact of the COVID-19 Pandemic on Healthcare-Associated Infections (HAIs)

To fully understand the impact of the COVID-19 pandemic on healthcare-associated infections (HAIs), general trends must be replaced by a critical, study-by-study comparison of clinical data recorded before and during the SARS-CoV-2 health crisis. The combination of severe viral pneumonias, prolonged invasive device utilization (such as mechanical ventilation and central venous catheters), and altered infection control protocols fundamentally shifted the incidence of multidrug-resistant organisms (MDROs) and fungal co-infections [45,46,47,48,49,50,51,52,53,54,55,56,57,58,59].

The incidence of critical Gram-negative and Gram-positive bacterial pathogens underwent severe changes during the pandemic waves compared to stable pre-pandemic baselines [45,46,47,48,49,50,51,52,53].

*Acinetobacter baumannii*: Multi-center epidemiological data show that carbapenem-resistant *Acinetobacter baumannii* (CRAB) experienced the most dramatic surge. According to national surveillance data published by the U.S. CDC, hospital-acquired CRAB cases surged by 78% in 2020 compared to 2019 [45]. In intensive care unit (ICU) settings, this increase was even more pronounced: a comprehensive multicenter study in Italy reported that the isolation rate of CRAB in healthcare-associated bacteremias jumped from a pre-pandemic baseline range of 0.3–8.0% up to 29.0–31.6% during the pandemic peaks [46].

*Pseudomonas aeruginosa:* The incidence of Pseudomonas aeruginosa tied to device-associated infections (DAIs) saw marked increases. In a comparative cohort analysis of critically ill patients, the relative risk of developing device-associated P. aeruginosa infection escalated significantly, with the overall hazard ratio for ventilator-associated pneumonia (VAP) driving a massive increase in Gram-negative dominance in the ICU during 2020–2021 compared to 2018–2019 [47].

Methicillin-Resistant *Staphylococcus aureus (MRSA)*: Hospital-acquired MRSA bacteremia rates, which had been steadily declining for a decade before 2019, reversed direction during the pandemic [48,49]. CDC surveillance registered a 13% increase in MRSA standardized infection ratios (SIR) in acute care hospitals in 2020 versus 2019 facilities [50]. On a regional level, European ICU monitoring data confirmed a statistically significant increase in MRSA prevalence, shifting from pre-pandemic baselines of 3.1–4.7% to pandemic levels of 5.0–5.2% [50].

*Klebsiella pneumoniae and Enterobacterales:* Extended-spectrum beta-lactamase (ESBL) and carbapenem-resistant Enterobacterales (CRE) infections rose sharply due to increased empirical antibiotic use [51,52,53]. A comparative study focusing on ICU patients revealed that ESBL positivity rates for K. pneumoniae stood at 61% in the pre-pandemic era (2018–2019) but spiked to 73% in general ICUs and reached an alarming 83% specifically within dedicated COVID-19 ICUs during the 2020–2021 pandemic period [51,52]. Concurrently, a point prevalence analysis across European tertiary hospitals identified a local HAI prevalence increase from 7.3% pre-pandemic to 9.0% during the pandemic, heavily driven by carbapenem-resistant K. pneumoniae lineages [53].

Emerging Fungal Pathogens and Co-infections. The disruption of standard stewardship and the widespread use of immunomodulatory therapies (e.g., corticosteroids, IL-6 inhibitors) for severe COVID-19 directly catalyzed a secondary epidemic of healthcare-associated fungal infections [54,55,56,57,58,59].

*Candida auris* presented an extraordinary clinical challenge due to its environmental persistence. National epidemiologic tracking from 2019 to 2021 demonstrated that clinical cases of C. auris in hospitalized patients rose by 60% in 2020 (757 cases) and surged by an additional 95% in 2021 (1,471 cases) compared to the pre-pandemic era [55]. Outbreaks were specifically clustered in COVID-19 ICUs, where personal protective equipment (PPE) reuse and overextended staff compromised standard contact precautions [55,56].

The broader incidence of central line-associated bloodstream infections (CLABSIs) caused by non-auris Candida species (such as Candida albicans and Candida glabrata) increased significantly. Systematic reviews pooling data from acute care settings identified a 27% higher risk (pooled OR: 1.27; 95% CI: 1.10–1.31) of acquiring fungal and device-associated bloodstream infections during the pandemic period compared to pre-pandemic baselines [57].

COVID-19-associated pulmonary aspergillosis (CAPA) emerged as a high-mortality clinical entity in mechanically ventilated patients. While invasive pulmonary aspergillosis was historically rare in non-immunocompromised ICU patients (incidence <1–2% pre-pandemic), multi-center retrospective trials identified that during the pandemic, the incidence of CAPA among critically ill COVID-19 patients requiring mechanical ventilation reached between 10% and 15%, representing an estimated five-to-seven-fold increase in hospital-acquired aspergillosis rates within the ICU population [58].

Regarding the use of devices, the increase in the incidence of sepsis caused by specific pathogens is mathematically correlated with variations in the usage rates of the devices themselves. Comparative tracking indicates that central line utilization grew by 10.7% and ventilator days grew by 12.9% during the pandemic. Consequently, the risk of developing a primary healthcare-associated bloodstream infection (HA-BSI) escalated significantly, shifting from a pre-pandemic baseline of 27.3 per 10,000 hospital days to 29.7 per 10,000 hospital days during the pandemic era (*p* = 0.006) [58]. This direct correlation emphasizes that the pandemic did not merely cause a generalized inflation of infection rates, but specifically multiplied device-associated sepsis risks across vulnerable patient cohorts [59].

## 4. Do COVID-19 Vaccines Play a Role in Limiting Healthcare-Associated Infections?

**Figure 1:**
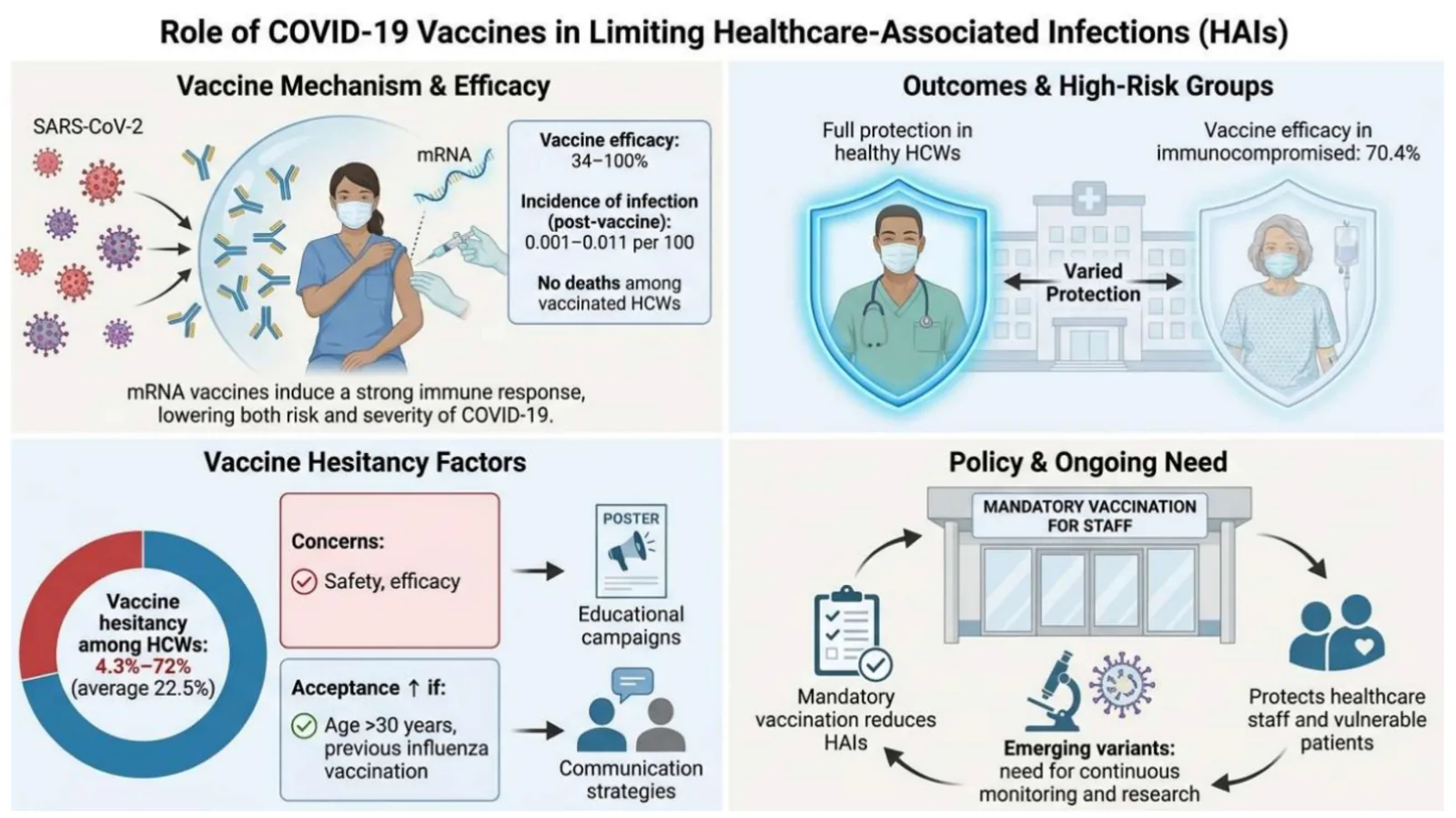
The role of COVID-19 vaccines in limiting healthcare-associated infections. This visual is organized into four structured panels, each focusing on different aspects of vaccine efficacy, the impact on healthcare workers, vaccine hesitancy, and the necessity for ongoing immunization efforts. The first panel depicts the mechanisms through which COVID-19 vaccines provide protection, emphasizing mRNA vaccines and their effectiveness against both symptomatic and severe infections. The second panel highlights the low incidence rates of post-vaccination infections among healthcare workers, detailing the reported statistics and showcasing the significant protection these vaccines afford. The third panel addresses the prevalent issue of vaccine hesitancy among healthcare professionals, incorporating relevant data and potential strategies for improvement, such as educational campaigns. Finally, the fourth panel discusses the implications for healthcare policies, including the importance of mandatory vaccination to enhance safety in healthcare environments.

COVID-19 vaccines play a crucial role in limiting healthcare-associated infections, particularly among healthcare workers, who have been identified as a high-risk population (Figure 1) [60,61]. Several studies have shown that mRNA vaccines provide effective protection against symptomatic and severe infections, significantly reducing the risk of hospitalization [60,61]. The incidence rate of post-vaccination infections is very low, with studies reporting a range of 0.001 to 0.011 per 100 at-risk individuals. However, despite these benefits, the rate of vaccine hesitancy among healthcare professionals is significant, indicating a need for stronger communication strategies. COVID-19 vaccines are essential tools for limiting healthcare-associated infections; evidence supports their high efficacy among healthcare workers, a group considered fundamental to COVID-19 control in healthcare settings [61,62]. One study revealed variable vaccine efficacy, with reported protection against infection ranging from 34% to 100%, and no deaths recorded among vaccinated workers in the analyzed studies. Data indicate that vaccination significantly reduces the risk of symptomatic infection, with strong evidence supporting mRNA vaccines, which show an extremely low incidence of post-vaccination infections: between 0.001 and 0.011 per 100 at-risk individuals [63]. Additionally, an analysis of vaccination among healthcare workers revealed a prevalence of vaccine hesitancy ranging from 4.3% to 72%, with an average of 22.51%. Concerns regarding vaccine safety and efficacy were identified as the primary reasons for hesitancy [64]. Conversely, vaccine acceptance was associated with factors such as being over 30 years of age and a history of influenza vaccination, suggesting that educational campaigns could improve vaccination rates [65]. Vaccine efficacy was found to be lower in immunocompromised individuals compared to healthy patients, highlighting the vulnerability of this population and the need for further research [66]. Nevertheless, the overall impact of vaccines in reducing the incidence of healthcare-associated infections is clear, justifying mandatory vaccination strategies for healthcare workers to curb transmission between patients and staff [67].

COVID-19 vaccines have demonstrated high efficacy in preventing symptomatic and severe infections among healthcare workers. A review reported that vaccine efficacy against infection ranges from 34% to 100%, with protection influenced by factors such as age and prior vaccination status [68]. A very low incidence of post-vaccination infections—ranging from 0.011 to 0.001 per 100 at-risk individuals—was observed, highlighting the efficacy of mRNA vaccines, particularly for healthcare professionals [69]. A systematic analysis indicated that vaccine efficacy is significantly lower among immunocompromised patients—showing an efficacy rate of 70.4% compared to full protection in healthy individuals—suggesting that vulnerable groups require special attention [66]. This underscores the importance of targeted vaccination strategies for at-risk populations [69,70,71,72]. In conclusion, COVID-19 vaccines play a significant role in limiting healthcare-associated infections, especially among healthcare workers. Evidence indicates very low incidence rates of post-vaccination infections and robust protection against severe forms of COVID-19. However, significant vaccine hesitancy among healthcare professionals raises concerns that require targeted interventions [69,70,71,72]. Mandatory vaccination policies and educational campaigns are crucial for protecting not only healthcare staff but also vulnerable patients in healthcare settings. Further research and continuous monitoring are essential to maintain the long-term efficacy of vaccination programs, particularly in the context of emerging variants [63,64].

## 5. What Was the Role of Antivirals Against COVID-19 in Preventing Healthcare-Associated Infections?

**Figure 2:**
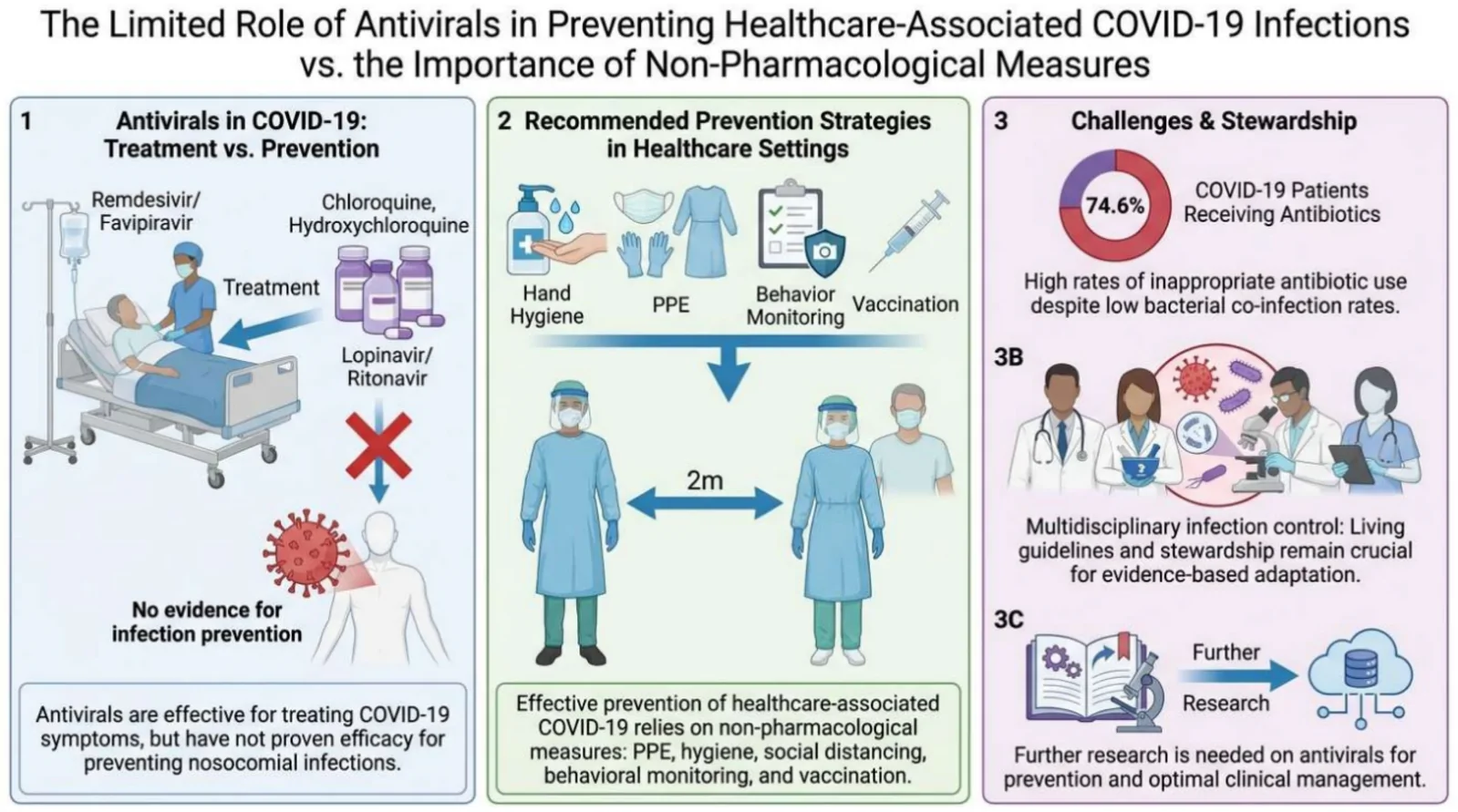
The role of antivirals in preventing healthcare-associated COVID-19 infections. The image clearly outlines the distinction between the treatment capabilities of antivirals and their inability to prevent infections in clinical settings. The first section illustrates the primary function of antivirals like remdesivir and favipiravir in managing COVID-19 symptoms rather than preventing infection. It also highlights the shift in focus toward non-pharmacological strategies such as social distancing, mask usage, and stringent hygiene practices as essential measures to mitigate the spread of the virus within healthcare environments. The second section provides an overview of current guidelines, which suggest that while antivirals can be effective in treatment, they are not sufficient for infection prevention, emphasizing the importance of interdisciplinary approaches and the need for ongoing research in this area.

The role of antivirals in preventing healthcare-associated COVID-19 infections has been quite limited, as their primary utility lies in treating the disease rather than preventing infection (Figure 2). Although antivirals such as remdesivir and favipiravir have proven effective in treating COVID-19 patients, there is no robust strategy for preventing infection in hospital settings [65,66,67]. Current recommendations focus more on non-pharmacological measures and clinical best-practice protocols to avoid SARS-CoV-2 infection. The role of antivirals in preventing healthcare-associated COVID-19 infections has been limited, given that they have primarily been used for symptom treatment and disease management. Data indicate that the use of antivirals like remdesivir has shown efficacy in treating COVID-19 patients, yet there is insufficient evidence to support their use for preventing nosocomial infections [65,66,67]. A detailed review of treatment protocols indicates that while antiviral therapies help manage COVID-19 cases, official recommendations focus primarily on non-pharmacological prevention measures—such as social distancing and hygiene—rather than the use of specific antivirals to prevent healthcare-associated infections [68]. One study examined antiviral treatment strategies, highlighting that while numerous drugs are under development and testing (e.g., favipiravir and remdesivir), their efficacy in directly preventing infection remains poorly defined [69]. Furthermore, guidelines from the Infectious Diseases Society of America have emphasized that the management of COVID-19 infections must continue to evolve, noting that there is currently no solid evidence supporting the use of antivirals to prevent healthcare-associated infections [70]. In the management of COVID-19, antivirals have primarily been considered for the treatment rather than the prevention of infection, despite drugs such as chloroquine, hydroxychloroquine, lopinavir/ritonavir, favipiravir, and remdesivir having been used based on observational studies and in vitro evidence, yet without clear efficacy in preventing infections [71]. COVID-19 treatment has involved the use of antiviral agents, but their application has not demonstrated a significant impact on reducing healthcare-associated infections within hospitals. A meta-analysis revealed that 74.6% of COVID-19 patients were prescribed antibiotics; thus, there was clearly a high incidence of inappropriate prescribing relative to the actual need for such therapies, given the low prevalence of bacterial co-infections [72]. Current guidelines—particularly those from the Infectious Diseases Society of America—have highlighted the need to develop evidence-based “living guidelines” to support clinicians in treating COVID-19. However, these recommendations focus more on treatment options than on preventive measures [73]. Such measures include the use of PPE, social distancing, and behavioral monitoring in healthcare settings, as also recommended during the pandemic in Germany [70]. Since healthcare-associated infections are a major concern in healthcare settings, it is essential to implement infection control protocols and rigorous hygiene practices rather than relying solely on antiviral therapies for prevention. Non-pharmacological strategies, alongside vaccines and mitigation measures, have proven more effective. Although antivirals offer therapeutic potential, a multidisciplinary approach to healthcare-associated infections is required. Many questions remain regarding the management and control of COVID-19 infections in healthcare environments. Therefore, encouraging further research into the efficacy of antivirals for infections in healthcare settings remains crucial [71,72,73]. In summary, the role of antivirals in preventing healthcare-associated infections in the context of COVID-19 has proven largely insufficient. While antiviral drugs have demonstrated efficacy in treating the disease, they do not contribute significantly to infection prevention in healthcare settings. Current policies and practices must prioritize non-pharmacological measures and infection monitoring, reserving the use of antivirals primarily for the treatment of infected patients [74,75,76]. Investments in research and clinical guidance remain essential for learning and adapting to this constantly changing landscape [77,78,79].

## 6. What Was the Role of Monoclonal Antibodies Used for COVID-19 in Preventing Healthcare-Associated Infections?

**Figure 3:**
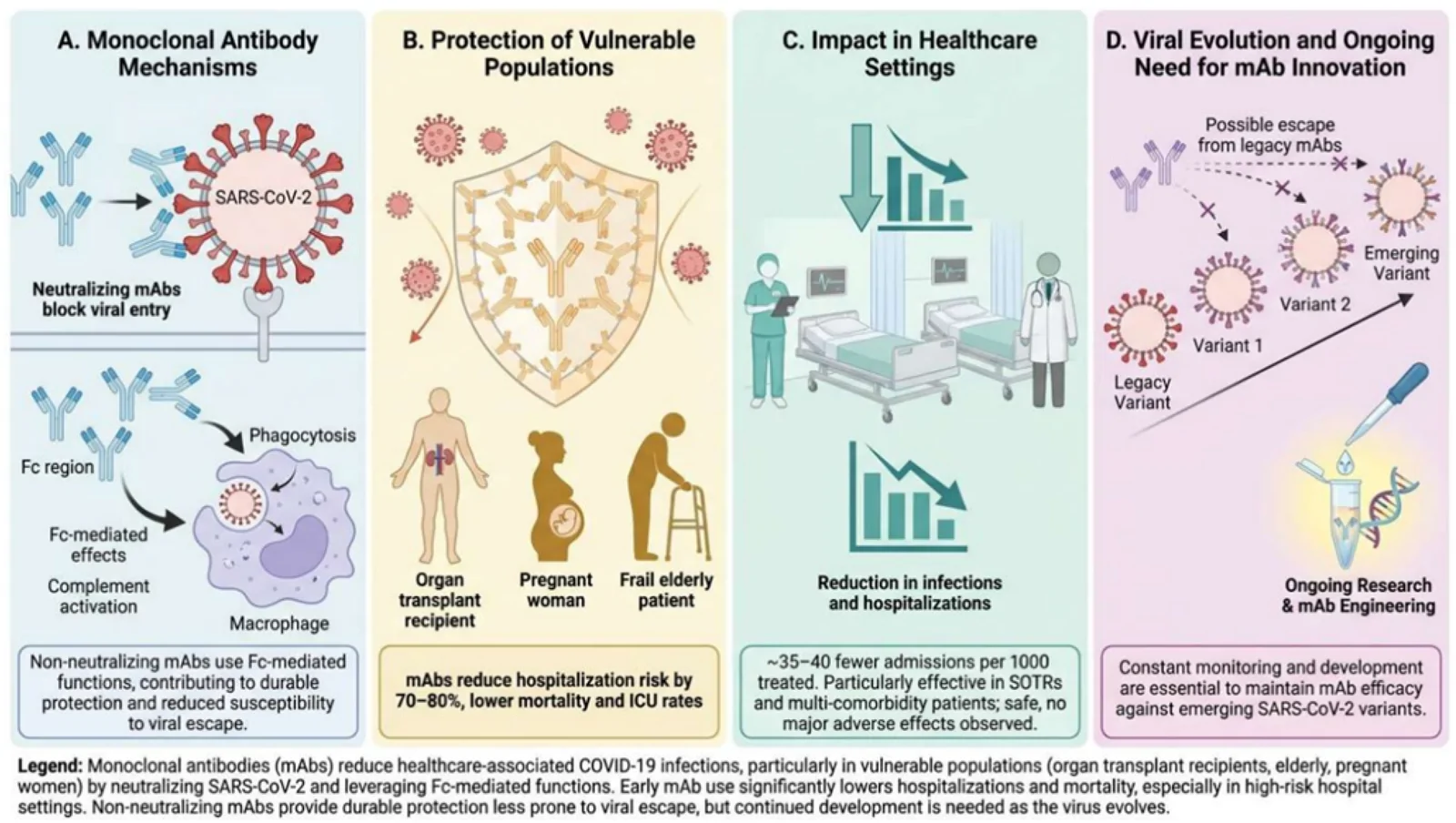
Role of monoclonal antibodies in preventing healthcare-associated COVID-19 infections. The image includes a detailed flowchart-style diagram organized into four distinct panels, each illustrating various aspects of monoclonal antibody mechanisms, their protection of vulnerable populations, their impact within healthcare settings, and the necessity for ongoing innovation in response to viral evolution. (**A**) The first panel depicts the mechanisms by which monoclonal antibodies interact with the SARS-CoV-2 virus, distinguishing between neutralizing and non-neutralizing antibodies. (**B**) The second panel focuses on at-risk populations such as organ transplant recipients and the protective effects of these antibodies against COVID-19 infections. (**C**) The third panel illustrates the overall impact of monoclonal antibodies in clinical environments, highlighting reductions in infections and hospital admissions. (**D**) Lastly, the fourth panel addresses the ongoing need for development due to viral variants and how research continues to evolve.

Monoclonal antibodies (mAbs) have been effectively used in the prevention and treatment of COVID-19 infection, demonstrating a reduction in healthcare-associated infections, particularly among vulnerable populations such as organ transplant recipients, frail patients, and pregnant women (Figure 3) [80,81,82,83,84,85]. Early use of mAbs in high-risk patients has led to a significant reduction in hospitalizations and mortality. Furthermore, non-neutralizing monoclonal antibodies have shown protective potential by bolstering the immune response [86]. However, it is crucial to consider the evolution of the virus and its ability to evade the effects of antibodies [87,88,89]. These specific antibodies were designed to recognize and neutralize the SARS-CoV-2 virus, helping to reduce both infection rates and disease severity. A systematic analysis indicated that mAbs can reduce the risk of COVID-19-related hospitalization by approximately 70–80% and have generally been associated with lower mortality rates and reduced intensive care utilization [90,91,92,93,94,95,96]. Particular importance has been attributed to non-neutralizing monoclonal antibodies, which have demonstrated potential in protecting against SARS-CoV-2 infection through Fc-mediated functions, such as complement activation and phagocytosis [97]. This offers a durable therapeutic option that may prove less susceptible to viral mutations compared to neutralizing mAbs. Additionally, a study highlighted that organ transplant recipients exhibit an excellent response to mAb treatment, showing no acute graft injury or serious adverse events associated with the use of these antibodies, thereby underscoring their safety and efficacy in the early treatment and prophylaxis of COVID-19 [98,99,100]. These results are particularly significant for the prevention of healthcare-associated infections, given that patients such as transplant recipients are already at higher risk of infectious complications; however, they are also relevant to the broader vulnerable population and to patients with multiple comorbidities hospitalized in clinical wards. Consequently, monoclonal antibodies have represented a major innovation in the care of COVID-19 patients, especially those at high risk. Various monoclonal antibodies—such as bamlanivimab and casirivimab—have been approved for emergency use, demonstrating significant evidence of efficacy in reducing severe outcomes associated with COVID-19 [98,99,100].

This is the only monoclonal antibody treatment that has demonstrated a significant reduction in hospitalizations, achieving an overall decrease of approximately 35–40 admissions per 1000 treated patients [98,99,100]. Another important aspect is that monoclonal antibodies may lose efficacy against emerging SARS-CoV-2 variants, suggesting that ongoing research and development are required to maintain the effectiveness of mAb therapies [101,102,103]. In this regard, monoclonal antibodies engineered for low affinity—such as non-neutralizing antibodies—have shown resilient protective potential by reducing susceptibility to viral mutations [104]. Solid organ transplant recipients (SOTRs) have been identified as an ideal population for monoclonal antibody treatment; immunosuppression and suboptimal vaccine responses render them particularly vulnerable to COVID-19 [105,106,107,108,109,110,111]. Several studies have demonstrated that the use of mAbs in these patients reduces not only the risk of infection but also the likelihood of severe adverse outcomes, such as the need for intensive care [105,106,107,108,109,110,111]. In conclusion, monoclonal antibodies have played a pivotal role in the prevention and treatment of COVID-19, particularly in healthcare settings and among vulnerable patient groups [112,113,114]. These therapies have been shown to reduce hospitalization and mortality rates, confirming their utility in disease control [115,116,117,118,119,120,121]. The virus’s continuous evolution implies that constant variant monitoring and the development of new monoclonal antibodies will remain crucial for managing healthcare-associated infections [122]. A proactive and informed approach to mAb administration can contribute to a significant reduction in the COVID-19 burden among vulnerable populations, thereby improving outcomes and the quality of care provided [123,124,125].

## 7. Conclusions

**Figure 4:**
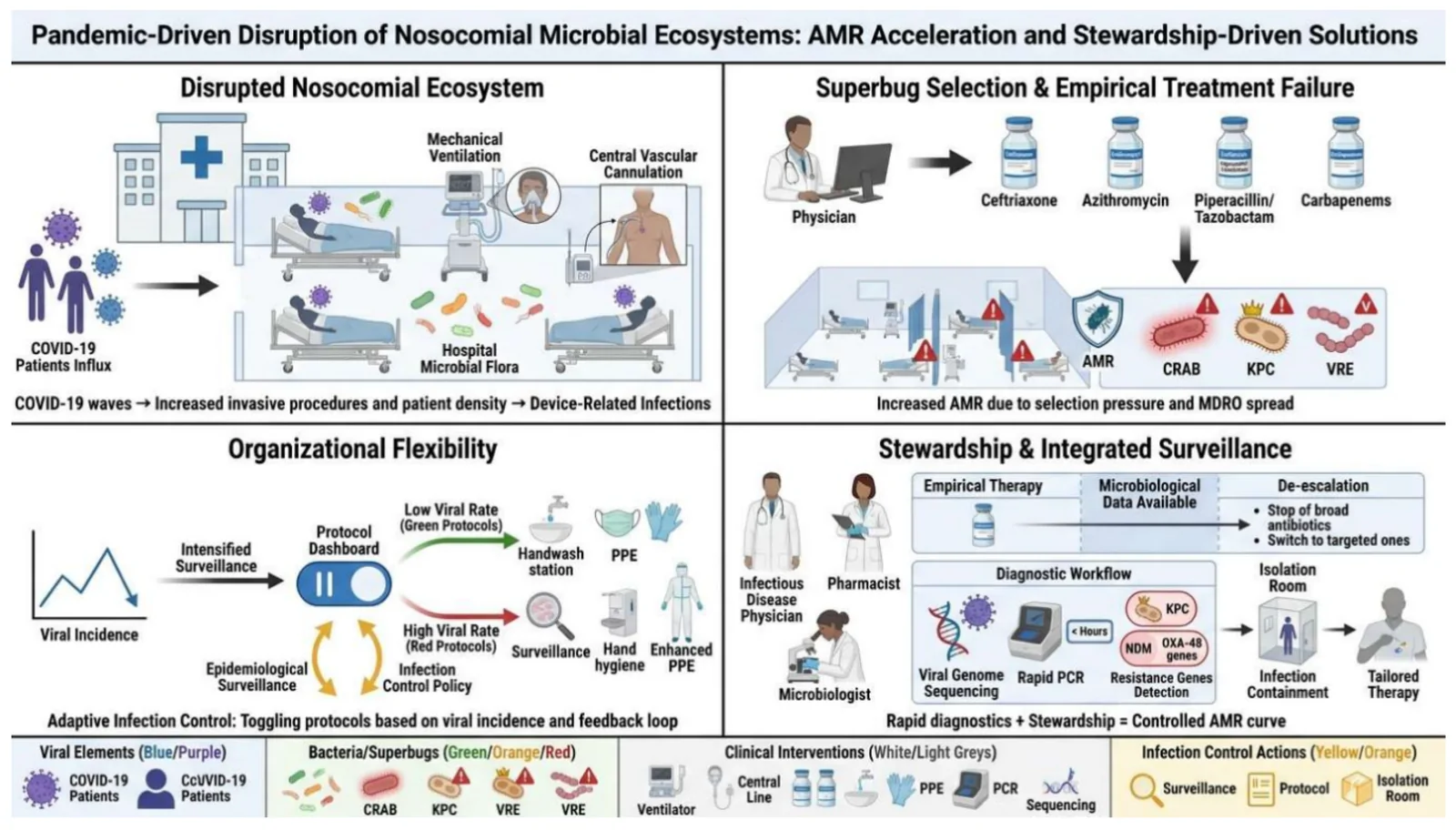
Future directions and solutions to avoid antibiotic resistance. The image consists of multiple panels, each illustrating critical aspects of this relationship, including the impact of COVID-19 on hospital procedures, the rise in antimicrobial resistance (AMR), and strategic responses for effective management. The first panel depicts how the pandemic disrupted traditional hospital ecosystems, leading to increased device-related infections due to a surge in invasive procedures. The second panel illustrates the impact of broad-spectrum antibiotic prescriptions on the emergence of multidrug-resistant organisms, showcasing the challenges posed by superbugs. The third panel emphasizes the need for adaptive organizational flexibility in infection control protocols in response to changing viral incidence rates. Finally, the fourth panel focuses on the role of antimicrobial stewardship and integrated surveillance in effectively managing AMR and improving patient outcomes.

In conclusion, the impact of COVID-19 on healthcare-associated infections (HAIs) represents a new frontier among the clinical challenges facing the healthcare sector. The emergence of resistant bacterial strains, the rising incidence of HAIs, and concerns regarding antibiotic management and usage are among the most pressing issues requiring attention. Healthcare strategies must be adapted to the incidence of viral infections and take into account the long-term implications of infection control policies. Promoting robust antibiotic stewardship policies and infection control practices is crucial for limiting complications associated with antibiotic resistance and improving patient outcomes both during and after the pandemic. To comprehensively and systematically address the complex interplay between viral pandemics, HAIs, and emerging antimicrobial resistance (AMR), the analysis must be structured around four fundamental clinical and organizational pillars (Figure 4).

- The New Epidemiological Frontier and the Nosocomial Ecosystem. The pressure exerted by COVID-19 epidemic waves disrupted traditional hospital microbiological ecosystems. The massive influx of patients with acute respiratory failure forced facilities to employ invasive procedures—such as prolonged mechanical ventilation and central vascular cannulation—on an unprecedented scale. This led to an increase in device-related infections. In this context, the virus acted not only as a primary pathogen but also as a systemic facilitator, prolonging hospital stays and multiplying opportunities for exposure to nosocomial microbial flora.
- Superbug Selection and Empirical Treatment Failure. During the early stages of the pandemic and subsequent variant-driven surges, the clinical overlap between viral pneumonia and potential bacterial coinfection prompted clinicians to prescribe broad-spectrum empirical antibiotics (such as ceftriaxone, azithromycin, piperacillin/tazobactam, and carbapenems) on a massive scale. Acceleration of antimicrobial resistance (AMR): This artificial selective pressure accelerated the emergence and inter-hospital spread of multidrug-resistant organisms (MDROs). Critical pathogens—such as carbapenem-resistant *Acinetobacter baumannii* (CRAB), carbapenemase-producing *Klebsiella pneumoniae* (KPC), and *vancomycin-resistant enterococci* (VRE)—found fertile ground to colonize the most vulnerable patients, drastically reducing last-resort therapeutic options.
- Organizational Flexibility. Unlike in the past, pandemic-related health measures must be adapted by updating infection control protocols; infection control policies can no longer remain static. They must be dynamically tailored to the epidemiological incidence of the virus. When viral infection rates rise, healthcare facilities must automatically activate intensified surveillance protocols. Healthcare management must therefore devise flexible organizational models aimed at maintaining high standards of care safety.
- The role of antimicrobial stewardship and surveillance in reversing the post-pandemic curve of bacterial resistance. It is crucial to implement rigorous antimicrobial stewardship programs supported by multidisciplinary teams (infectious disease specialists, clinical pharmacists, and microbiologists). These programs must mandate the timely de-escalation of empirical therapy as soon as microbiological data become available. Integration of virology and bacteriology: proactive management of vulnerable patients requires combining viral genomic surveillance (to map emerging variants and guide the use of appropriate monoclonal antibodies) with rapid molecular diagnostic techniques for bacteria. Only by identifying bacterial resistance genes (such as KPC, NDM, and OXA-48) within hours of admission is it possible to isolate the patient, halt horizontal transmission within wards, and tailor therapy.

In summary, the fundamental lesson of the pandemic is that managing a viral crisis cannot be divorced from preserving our antibiotic arsenal and rigorously controlling secondary bacterial infections. The sustainability of modern medicine and the safety of oncology, transplant, and critically ill patients depend entirely on the ability of healthcare systems to combine virological preparedness with a robust defense against antimicrobial resistance.

## Figures and Tables

**Table 1:** Summary of bacterial and fungal HAIs during COVID-19. The clinical manifestations, pandemic-specific risk factors, and outbreak mechanisms of these prominent bacterial and fungal HAIs.

Pathogen	Clinical Disease	Key Pandemic Drivers	Outbreak Mechanism
MRSA/ Staphylococci	Bacteremia, BSI, Pneumonia	Catheterization, antibiotic overuse	Direct contact, poor skin barriers
*P. aeruginosa*	VAP, Device infections	Long ventilation, ICU understaffing	Biofilms on respiratory equipment
*A. baumannii* (CRAB)	Severe VAP, Bloodstream	High patient-staff ratios, poor cleaning	High environmental persistence
MDR Enterobacterales	Bacteremia, UTIs	Selection pressure, screening lapses	Cross-contamination in cohorts
*Candida auris*	Candidemia, Wound inf.	Staff burnout, poor disinfectants	PPE shortages and reuse
CAM (Mucormycosis)	Rhino-orbital, Pulmonary	Corticosteroids, Diabetes, Hypoxia	Spore inhalation, angioinvasion

**Table 2:** Factors altering HAI rates during COVID-19. This table explicitly categorizes and balances the opposing factors that contributed to the increase or decrease in HAIs, clearly demonstrating how the systemic, clinical, and operational pressures of the COVID-19 pandemic directly drove these epidemiological shifts.

Impact on HAIs	Contributing Factor	Specific Pandemic Driver	Direct Link?
INCREASE	Corticosteroids	Immunosuppression favoring CAM and fungal infections	Yes (Clinical protocol)
	PPE Shortages/Reuse	Extended wear turned gowns/gloves into vectors	Yes (Supply collapse)
	ICU Staff Burnout	High patient-nurse ratios caused hygiene lapses	Yes (System overload)
	Antibiotic Overuse	Mass empirical treatment selected MDR bacteria	Yes (Co-infection fear)
	Surveillance Lapses	Suspended screening allowed undetected spread	Yes (Resource diversion)
DECREASE	Masking & Isolation	Strict airborne isolation reduced respiratory viruses	Yes (Mitigation policies)
	Initial Hand Hygiene	Intense early-phase focus lowered contact infections	Yes (Early response)

## Data Availability

No new data were created or analyzed in this study. Data sharing is not applicable to this article.
